# Fractal dimension of the aortic annulus: a novel predictor of paravalvular leak after transcatheter aortic valve implantation

**DOI:** 10.1007/s10554-022-02657-1

**Published:** 2022-06-24

**Authors:** Georg Stachel, Mohamed Abdel-Wahab, Suzanne de Waha-Thiele, Steffen Desch, Hans-Josef Feistritzer, Mitsunobu Kitamura, Serdar Farhan, Ingo Eitel, Thomas Kurz, Holger Thiele

**Affiliations:** 1 Department of Internal Medicine/Cardiology and Leipzig Heart Institute, Heart Center Leipzig at University of Leipzig, Leipzig, Germany; 2 Department of Cardiac Surgery, Heart Center Leipzig at University of Leipzig, Strümpellstr. 39, 04289 Leipzig, Germany; 3 University Clinic Schleswig-Holstein, University Heart Center Luebeck, Lübeck, Germany; 4 The Zena and Michael A. Wiener Cardiovascular Institute, Icahn School of Medicine at Mount Sinai, New York, NY USA; 5 DZHK (German Centre for Cardiovascular Research), Partner Site Hamburg/Luebeck/Kiel, Lübeck, Germany

**Keywords:** Transcatheter aortic valve implantation, Aortic stenosis, Computerized tomography (CT), Fractal dimension, Automated image analysis

## Abstract

To evaluate the prognostic relevance of aortic annulus (AA) and left ventricular outflow tract (LVOT) Fractal dimension (FD). FD is a mathematical concept that describes geometric complexity of a structure and has been shown to predict adverse outcomes in several contexts. Computed tomography (CT) scans from the SOLVE-TAVI trial, which, in a 2 × 2 factorial design, randomized 447 patients to TAVI with the balloon-expandable Edwards Sapien 3 or the self-expanding Medtronic Evolut R, and conscious sedation or general anesthesia, were analyzed semi-automatically with a custom-built software to determine border of AA and LVOT. FD was measured by box counting using grid calibers between 0.8 and 6.75 mm and was compared between patients with none/trivial and mild/moderate paravalvular regurgitation (PVR). Overall, 122 patients had CT scans sufficient for semi-automatic PVR in 30-day echocardiography. PVR was none in 65(53.3%) patients, trace in 9(7.4%), mild in 46(37.7%), moderate in 2(1.6%) and severe in 0 patients. FD determined in diastolic images was significantly higher in patients with mild/moderate PVR (1.0558 ± 0.0289 vs. 1.0401 ± 0.0284, p = 0.017). Annulus eccentricity was the only conventional measure of AA and LVOT geometry significantly correlated to FD (R = 0.337, p < 0.01). Area under the curve (AUC) of diastolic annular FD for prediction of mild/moderate PVR in ROC analysis was 0.661 (0.542–0.779, p = 0.014). FD shows promise in prediction of PVR after TAVI. Further evaluation using larger patient numbers and refined algorithms to better understand its predictive performance is warranted.

**Trial Registration:** www.clinicaltrials.gov, identifier: NCT02737150, date of registration: 13.04.2016.

**Supplementary Information:**

The online version contains supplementary material available at 10.1007/s10554-022-02657-1.

## Introduction

Paravalvular regurgitation (PVR) remains an important drawback of transcatheter aortic valve implantation (TAVI) with negative prognostic implications for the patient [1–3]. Apart from procedural causes, its incidence depends on sealing zone anatomy, quantified for example by measures of calcium protrusion into the lumen [4], semiquantitatively graded calcium bulks [5] or landing zone eccentricity or nontubularity [6, 7]. However, these predictors could not be substantiated in all studies, and an objective quantification of ‘disadvantageous anatomy’ has not yet been developed. It is conceivable that the use of measurements derived from Euclidean geometry such as eccentricity or simple asymmetry underestimates the complexity of the surface of aortic valve and perivalvular structures, which result from intricate growth and degeneration processes. Fractal geometry might be useful to describe and analyze these complex structures.

**Fig. 1:**
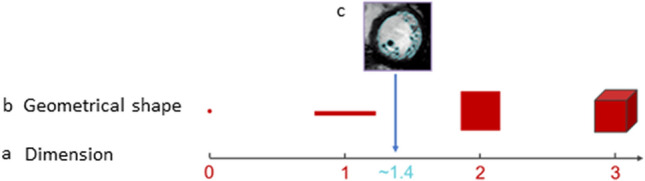
Fractal dimension. Row **a** dimension. Row **b** geometrical shapes with integer Euclidean dimensions. **c** Complex shape of the left ventricular endocardial border with a FD of approximately 1.4, i.e., between one-dimensional line and two-dimensional square (Magnetic resonance image reprinted by permission from Springer Nature [11])

Generally, a fractal is a complex set or shape which can be generated by recursively repeating an algorithm [8] and thereby ‘growing’ a structure which is self-similar, i.e., showing details at a certain scale which are similar to those observed at larger or smaller scales [9]. Many natural structures appear to be fractal, such as coastlines, clouds or plant structures [10], folds of brain or bowel [9], branching patterns of coronary vessels or the endocardial boundaries of left ventricular myocardium (Fig. 1) [11].

Fractal dimension (FD) can be thought of as a sum measure of complexity of a structure too rough or irregular to be entirely defined by Euclidean geometry [12]. FD for a line which is not straight, but space-filling will be between 1 (the dimension of a straight line) and 2 (the dimension of a plane), and will tend to 2 with increasing complexity and space-filling capacity of the line. FD can be determined objectively and has been shown to differ in health and disease in several cardiac conditions: The FD for left ventricular trabeculae in 2D cardiac magnetic resonance images differed in healthy persons and patients with left ventricular non-compaction cardiomyopathy [13] and was prognostic in hypertrophic cardiomyopathy [14], and fractal indices of heart rate variability have demonstrated prognostic capability for sudden cardiac death [15, 16].

We hypothesized that FD of the annular or left ventricular outflow tract (LVOT) endocardial border as a measure of complexity and ‘roughness’ of the anatomy could predict PVL incidence after TAVI. We evaluated this hypothesis using data from the randomized CompariSon of secOnd-generation seLf-expanding Versus balloon-expandable valves and gEneral versus local anesthesia in Transcatheter Aortic Valve Implantation (SOLVE-TAVI) trial [17]. To our knowledge, this is the first application of this measure in the context of TAVI.

## Methods

### The SOLVE-TAVI trial

The SOLVE-TAVI trial (ClinicalTrials.gov identifier: NCT02737150) was a 2 × 2 factorial randomized trial comparing the balloon-expandable Edwards Sapien 3 (BEV) to the self-expanding Medtronic Evolut R (SEV) and general anesthesia to conscious sedation in a high-risk cohort with symptomatic severe aortic stenosis. Details of the trial design and the results of the primary outcome have been published elsewhere [17, 18]. The ethics committees of all participating centers and national regulatory authorities approved the trial and the participants gave written informed consent.

### Assessment of outcomes

PVR was assessed by echocardiography at 30 days and graded as none/trace (reported as a common category), mild, moderate, or severe, according to VARC-2 [19] by local center and a blinded core lab at the Heart Center Leipzig at the University of Leipzig. We also assessed a composite endpoint comprising all-cause death, stroke, moderate or severe PVR, annular rupture and permanent pacemaker implantation (PPI) at 30 days. The components were defined according to VARC-2 criteria [19].

### Manual analysis of multidetector row computed tomography images

Multidetector computed tomography (MDCT) was performed as per site-specific protocol and analyzed at the study core lab at the Heart Center Leipzig using dedicated software (3mensio, Pie Medical Imaging, Maastricht, The Netherlands).

As described previously [20], the annular plane was defined by the 3 basal hinge points of the aortic cusps in the LVOT, and the LVOT plane was determined to be 5 mm below the annular plane. Eccentricity was calculated as 100*(maximum diameter − minimum diameter)/maximum diameter. LVOT non-tubularity was calculated as (annulus area − LVOT area)/annulus area. LVOT and annular area and circumference as well as minimal and maximal diameters were determined by an observer blinded for outcome and automatically determined measurements.

### Semiautomatic analysis of MDCT images

**Fig. 2:**
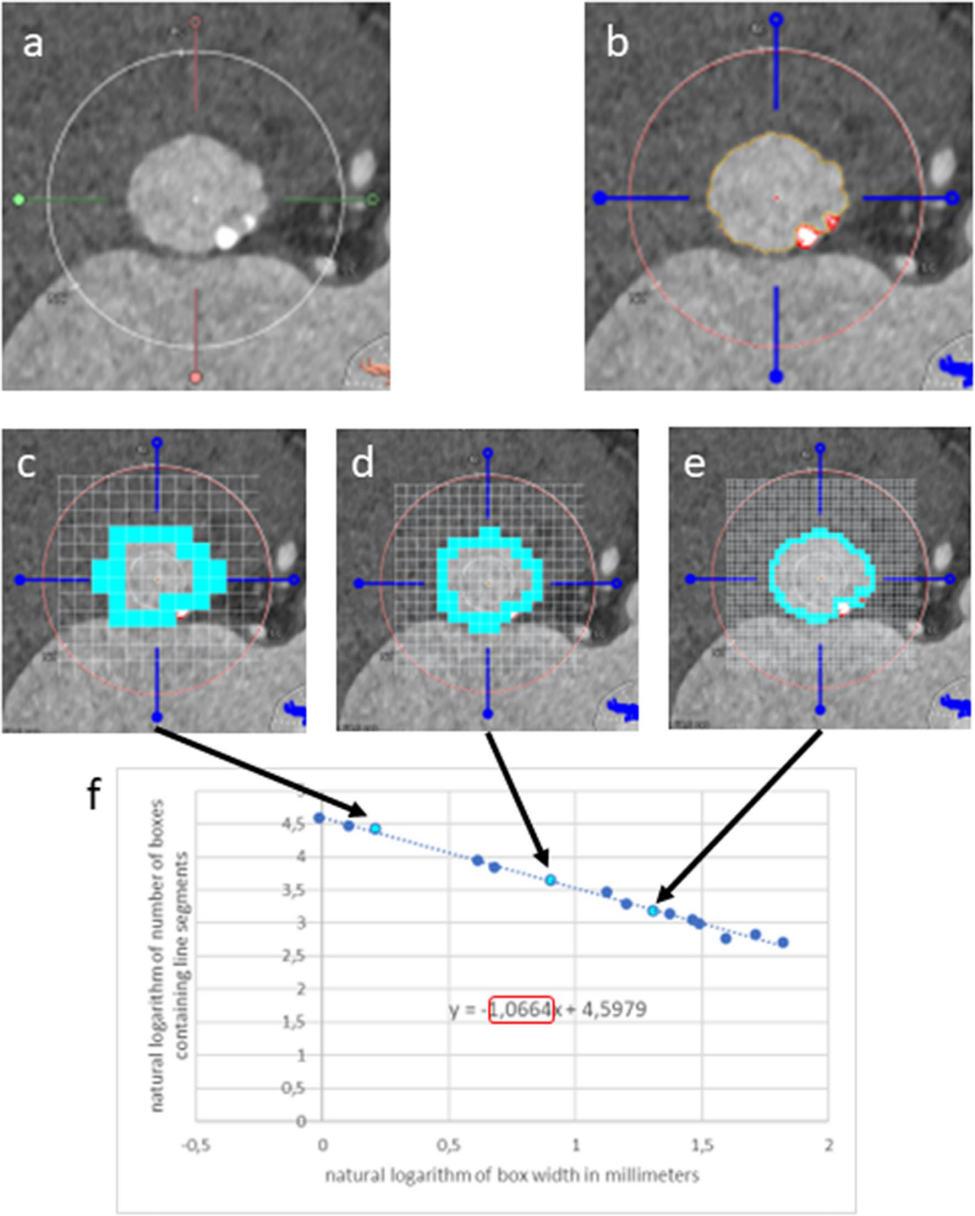
Analysis of MDCT images and determination of FD by box counting. **a** MDCT image of aortic annulus as exported from 3mensio. White arrow denotes ‘compass’ ring with true diameter calibration from 3mensio. **b** Segmented image, border line of the aortic annulus is shown in yellow. **c**–**e** Examples of FD determination by box counting. The border line is overlaid by grids of several different calibers (white lines). The number of boxes containing segments of the border line (light blue boxes) are counted for each grid. **f** The natural logarithm of the number of boxes is plotted against the natural logarithm of box width. Light blue dots are the datapoints generated from panels **c**–**e**, dark blue dots are the datapoints generated by box counting using different box calibers than displayed in panels c-e. FD of the border line is the absolute value of the slope of the fitting line (red frame)

A patient was deemed suitable for semiautomatic analysis of the images with the current segmentation algorithm if MDCT if luminal attenuation was above 300 Hounsfield units. LVOT and annulus images for each available cardiac phase were then exported from 3mensio in Tagged Image File (TIF) format (Fig. 2A) and loaded into a program custom-built using C# code in VisualStudio 2017 (Microsoft Corporation, Redmond, WA, USA), where the border of the aortic annulus was determined semi-automatically by detection of the change in pixel brightness between lumen and annular tissue, or calcium, respectively (Fig. 2B). In case of incorrect detection or fragmentation of the detected edge, the detected line was corrected manually. The source code of the program for determination of the borderline and its FD has been made publicly available at the Harvard Dataverse repository and can be accessed at https://doi.org/10.7910/DVN/Q5HXK5.

### Determination of FD

FD of the annulus and LVOT border line was determined by automated box counting [11] in the same custom-built program using a fixed grid with grid calibers between 0.8 and 6.75 mm, and determining the number of boxes containing a part of the border line for each grid caliber (Fig. 2C–E).

The conversion factor between pixels and mm for each image was determined by using the diameter of the ‘compass’ ring added to the image in 3mensio.

The natural logarithm of box caliber was plotted against the natural logarithm of the number of boxes of the respective caliber containing a part of the border line. The box counting dimension was determined as the absolute value of the slope of the fitting line constructed using the least ordinary squares method (Fig. 2F).

### Statistical analyses

Characteristics of both groups are reported as absolute and relative frequencies for categorical variables and median and interquartile range (IQR) for ordinal variables and continuous variables which were not normally distributed. The denominator of proportions may differ because of missing values which were not imputed. Categorical variables were compared by Chi-Square test and Fisher’s exact test where appropriate. Distribution normality was assessed by Shapiro–Wilk test. Variables which were not normally distributed were compared by Mann–Whitney-U-test. Pearson’s correlation coefficient was calculated for FD and variables taken from the literature as influencing PVR incidence. Receiver-operator-characteristic (ROC) curves were calculated, and the AUC and its 95% confidence interval was determined. Cut-off for optimal sensitivity and specificity was determined by Youden’s method.

To assure annulus and LVOT segmentation was accurate, Cronbach’s alpha and intraclass correlation coefficient for absolute agreement were determined between the area of the automatically segmented structure and the respective manually segmented annuli and LVOT.

We defined significance level at 5% for two-tailed testing. Data analysis was performed using SPSS version 22 (IBM, Armonk, New York, USA).

## Results

### Baseline and procedural characteristics

**Fig. 3:**
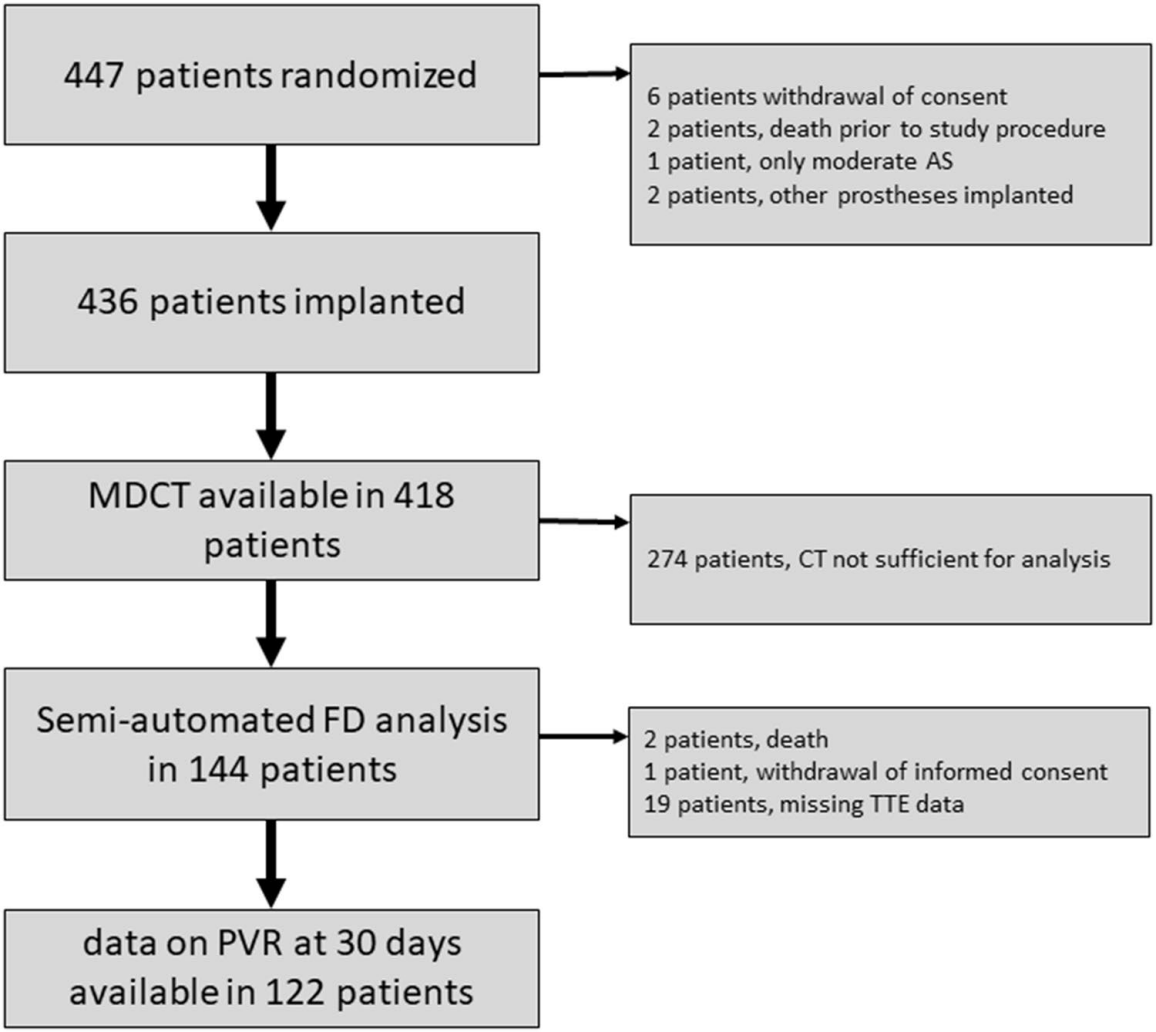
Patient flow chart

The SOLVE-TAVI trial randomized a total of 447 patients between April 2016 and April 2018 (Fig. 3). Of these, 438 patients underwent TAVI. MDCT was available in 418 of these patients. In 144 patients, MDCT showed a luminal attenuation of more than 300 Hounsfield units and hence was sufficient for segmentation of the aortic annulus with the current algorithm. Of those 144 patients, 69 (47.9%) received a balloon-expandable valve prosthesis (BEV, Sapien 3, Edwards Lifesciences, Irvine, CA, USA) and 75 patients (52.1%) received a self-expanding valve prosthesis (SEV, Evolut R, Medtronic, Minneapolis, MN, USA).

Systolic MDCT images were available in 44 patients (30.5%), diastolic MDCT images in 48 patients (33.3%) and images in both phases in 52 patients (36.1%).

Data on PVR after 30 days were available in 122 patients (84.7%). Of those, 65 (53.3%) had no PVR, 9 (7.4%) had trace PVR, 46 (37.7%) had mild PVR and 2 patients (1.6%) had moderate PVR. There was no case of severe PVR.

Baseline characteristics are shown in Table 1. There were no significant differences in clinical baseline characteristics between patients with no/mild PVR and patients with moderate/severe PVR.

**Table 1:** Baseline clinical and echocardiographic characteristics

	No/trace PVR	Mild/moderate PVR	p =
Number of patients	74	48	
Implanted prosthesis			0.097
BEV	40 (54.1%)	18 (37.5%)	
SEV	34 (46.0%)	30 (62.5%)	
Age (years); median (IQR)	82.0 (78.0–85.0)	81.5 (79.0–86.0)	0.618
Male sex; n/total (%)	33 (44.5.2%)	22 (45.8%)	1.0
Risk scores			
STS-PROM (%); median (IQR)	4.50 (3.40–8.91)	4.70 (3.03–7.53)	0.510
Log. EuroSCORE I (%); median (IQR)	12.60 (8.11–18.80)	13.00 (8.40–24.50)	0.583
EuroSCORE II (%); median (IQR)	3.70 (2.45–6.10)	4.45 (2.53–6.15)	0.485
Body mass index (kg/m^2^); mean ± SD	26.76 ± 4.86	26.93 ± 3.55	0.781
Arterial hypertension; n/total (%)	68 (93.2%)	42 (87.5%)	0.341
Diabetes mellitus; n/total (%)	26 (36.1%)	18 (37.5%)	1.0
Hyperlipoproteinemia; n/total (%)	42 (58.3%)	27 (56.3%)	0.852
Any coronary artery disease; n/total (%)	36 (54.6%)	21 (43.8%)	0.242
1-vessel disease; n/total (%)	16 (24.2%)	5 (10.4%)	
2-vessel disease; n/total (%)	10 (15.2%)	6 (37.5%)	
3-vessel disease; n/total (%)	10 (15.2%)	10 (20.8%)	
Left main disease; n/total (%)	0 (0%)	0 (0%)	
Prior PCI; n/total (%)	23 (31.5%)	13 (27.1%)	0.686
Prior CABG; n/total (%)	6 (8.2%)	2 (4.2%)	0.476
Prior myocardial infarction; n/total (%)	8 (11.0%)	4 (8.3%)	0.762
Peripheral arterial disease; n/total (%)	9 (12.3%)	4 (8.3%)	0.562
Prior stroke; n/total (%)	8 (11.1%)	7 (14.6%)	0.585
Any renal insufficiency; n/total (%)	54 (74.0%)	39 (81.3%)	0.501
Stages II and IIIa; n/total (%)	36 (49.3%)	23 (48.0%)	
Stages IIIb, IV and V; n/total (%)	18 (24.7%)	16 (33.3%)	
Atrial fibrillation; n/total (%)	33 (45.2%)	22 (45.8%)	1.0
Prior pacemaker/ICD/CRT; n/total(%)	4 (5.5%)	3 (6.4%)	1.0
COPD; n/total (%)	16 (21.9%)	8 (16.7%)	0.642
Pulmonary hypertension; n/total (%)	37 (51.4%)	28 (59.6%)	0.452
New York Heart Association Class			
I; n/total (%)	3 (4.1%)	6 (13.0%)	
II; n/total (%)	18 (24.7%)	9 (19.6%)	
III; n/total (%)	48 (65.8%)	28 (60.9%)	
IV; n/total (%)	4 (5.5%)	3 (6.5%)	
Baseline echocardiographic findings			
Aortic valve area (cm^2^); median (IQR)	0.70 (0.60–0.90)	0.60 (0.60–0.88)	0.355
Mean aortic valve gradient (mmHg); median (IQR)	43.0 (34.0–49.0)	36.5 (31.3–45.0)	0.124
Left ventricular ejection fraction			
> 55%; n/total (%)	48 (66.7%)	26 (55.3%)	
45–55%; n/total (%)	19 (26.4%)	14 (29.8%)	
35–44%; n/total (%)	3 (4.2%)	2 (4.3%)	
< 35%; n/total (%)	2 (2.8%)	5 (10.6%)	

*IQR* interquartile range, *ICD* implantable cardioverter-defibrillator, *CRT* cardiac resynchronization therapy device, *STS-PROM* Society of Thoracic Surgeons—Predicted Risk of Mortality, *PCI* percutaneous coronary intervention, *CABG* coronary artery bypass graft, *COPD* chronic obstructive pulmonary disease

### MDCT analysis

**Fig. 4:**
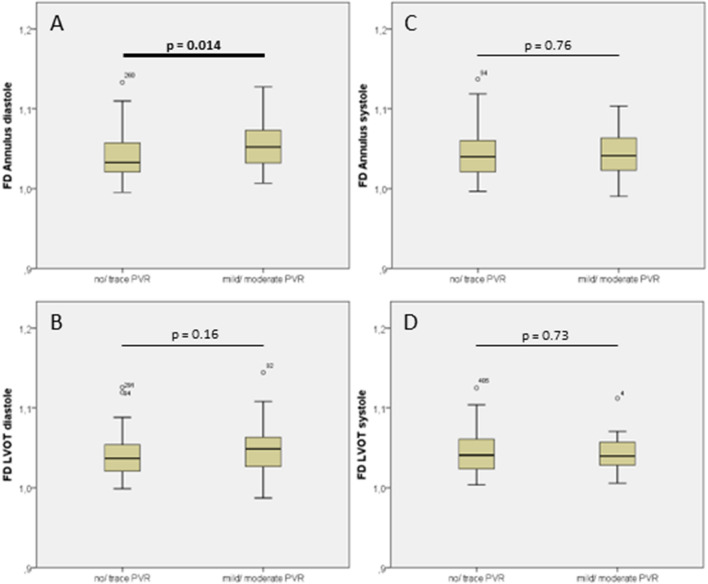
FD in patients with and without mild/ moderate PVR. **a** FD of annulus in diastole **b** FD of LVOT in diastole. **c** FD of annulus in systole. **d** FD of LVOT in systole

Annular FD determined in diastolic images was significantly higher in patients with mild or greater PVR than in patients with none or trace PVR [no/trace PVR 1.0327 (1.0208–1.0575) vs. mild/moderate PVR 1.0522 (1.0320–1.0731), p = 0.014, Fig. 4, Panel A]. There was no significant difference in annular FD determined in systolic images [1.0399 (1.0210–1.0604) vs. 1.0413 (1.0231–1.0636), p = 0.755, Fig. 4B] or in LVOT FD either in diastole [1.0368 (1.0204–1.0543) vs. 1.0487(1.0266–1.0634), p = 0.161, Fig. 4C] or in systole [1.0410 (1.0235–1.0609) vs. 1.0397 (1.0283–1.0571), p = 0.733, Fig. 4D].

When examining BEV and SEV separately, there was significant difference after BEV implantation in diastolic annular FD [no/trace PVR 1.0313 (1.0238–1.0537) vs. mild/moderate PVR 1.0511 (1.0381–1.0862), p = 0.026], but no significant difference between the PVR groups was found in LVOT or systolic dimensions [annulus: 1.0381 (1.0176–1.0558) vs. 1.0413 (1.0209–1.0628), p = 0.509; LVOT systolic 1.0409 (1.0216–1.0609) vs. 1.0504 (1.0365–1.0596), p = 0.387; LVOT diastolic 1.0346 (1.0185–1.0525) vs. 1.0492 (1.0378–1.0542), p = 0.105, no/trace PVR vs. mild/moderate PVR, respectively] or in patients after SEV implantation [annulus/diastolic 1.0334 (1.0168–1.0589) vs. 1.0554 (1.0235–1.0731), p = 0.163; LVOT/diastolic 1.0368 (1.0271–1.0673) vs. 1.0412 (1.0266–1.0672), p = 0.862; annulus/systolic 1.0434 (1.0275–1.0691) vs. 1.0422 (1.0249–1.0767), p = 0.751; LVOT/systolic 1.0429 (1.0268–1.0631) vs. 1.0356 (1.0279–1.0487), p = 0.301; no/trace PVR vs. mild/moderate PVR, respectively].

**Fig. 5:**
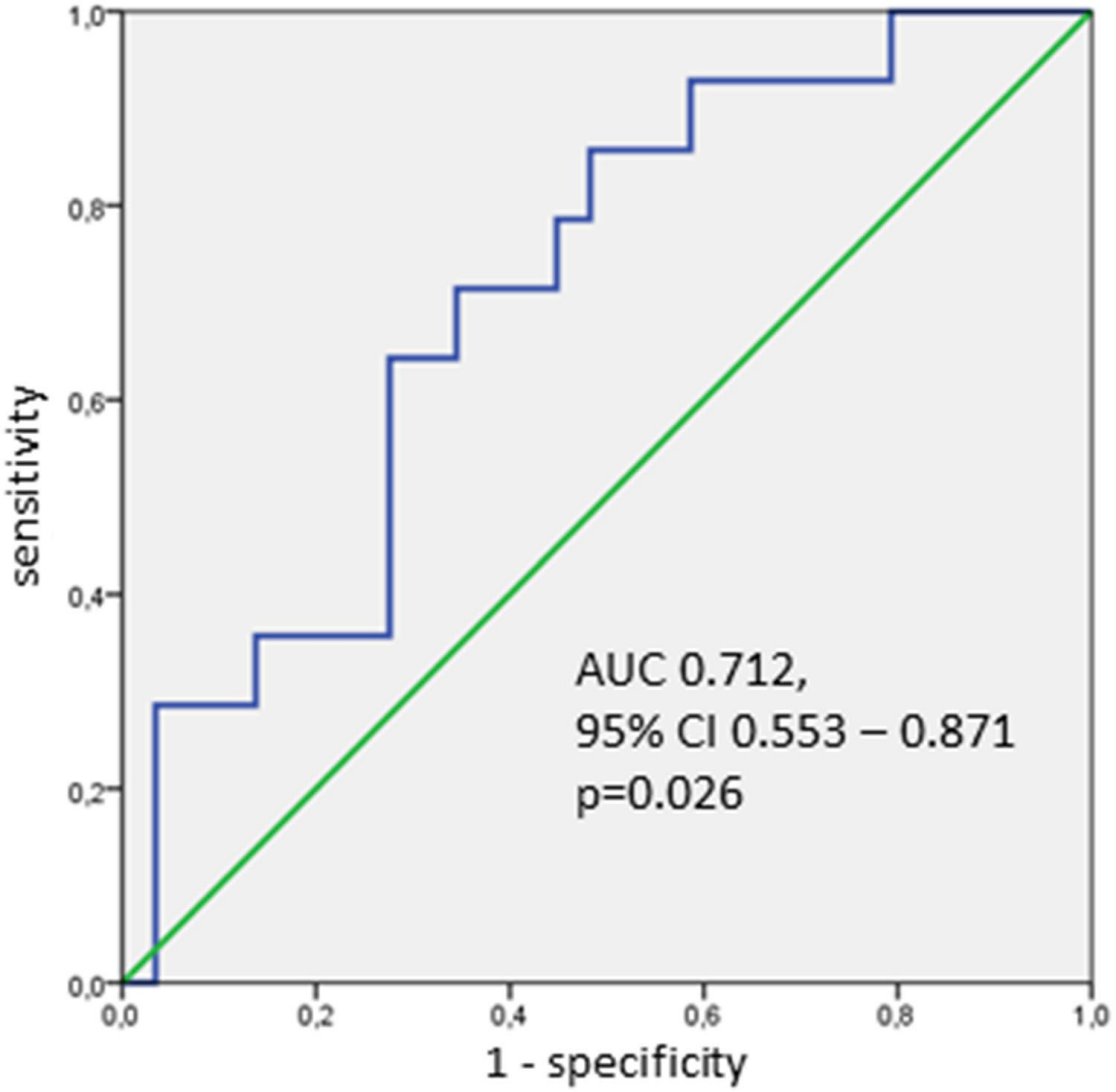
Predictive power of FD. ROC analysis of prediction of mild or more PVR after BEV treatment by FD of annulus. *CI* confidence interval, *AUC* area under the curve

The AUC of diastolic annular FD in ROC analysis was 0.661 (IQR 0.542–0.779, p = 0.01, Fig. 5). Optimal sensitivity (60.6%) and specificity (70.0%) was found at a FD level of 1.0502.

Concerning the composite endpoint of all-cause death, stroke, moderate or severe PVR, PPI and annulus rupture, we found no significant differences in FD between groups [1.0368 (1.0202–1.0569) vs. 1.0510 (1.0286–1.0611), p = 0.1, Supplementary Table S1].

There was strong correlation between the automatically determined area of the annulus or LVOT and the respective manually determined area for annulus in diastole [Cronbach’s Alpha 0.977, intraclass correlation coefficient for absolute agreement (ICAA) 0.969, 95% confidence interval (CI) 0.923–0.985, p < 0.01], annulus in systole (Cronbach’s alpha 0.964, ICAA 0.947, 95% CI 0.831–0.976, p < 0.01) as well as for LVOT area in diastole (Cronbach’s alpha 0.984, ICAA 0.983, 95% CI 0.972–0.989, p < 0.01) and systole (Cronbach’s alpha 0.986, ICAA 0.980, 95% CI 0.934–0.991, p < 0.01).

When examining the correlation of FD to other measures of annular and LVOT geometry, we found only weak positive correlations of FD to annulus eccentricity (R = 0.337, p = 0.001) and the semiquantitative measure of LVOT calcification (R = 0.207, p = 0.045). We did not find a significant correlation to other anatomical parameters identified as predictors of PVL after TAVI by the existing literature (Table S2).

## Discussion

We determined FD by semi-automated analysis of a subset of MDCT images from the SOLVE-TAVI trial and found that FD of the aortic annulus in diastole was significantly higher in patients who developed mild or greater PVR after TAVI. We did find only weak correlations to conventional anatomical predictors of mild or more PVR after TAVI. To the best of our knowledge, this is the first proof of concept concerning the application of FD in the prediction of outcomes after TAVI.

### Rationale

There has been extensive research into anatomical causes of PVR after TAVI. Almost always, measures of asymmetry or unevenness of LVOT or annulus such as LVOT or annular eccentricity [6], leaflet asymmetry, LVOT non-tubularity [7], calcium asymmetry [21] or protrusion of calcium -rather than absolute calcium load- have been implicated. As the results were mixed and in part contradictory, a more comprehensive measure such as FD may be useful.

Fractal dimension is no ‘size’ of the annulus but rather an unitless number describing its geometrical complexity [11]. In our analysis, there has been a weak correlation between eccentricity as well as LVOT calcium, and FD, yet explaining only part of FD extent. Large foci of calcium would most likely cause indentations of the border line, thereby increasing FD.

Some anatomies of the LVOT have also been found to increase complications such as stroke [22], PVR [6], PPI [23] or annular rupture [24], hence assessment of a composite clinical endpoint is also reasonable.

Moderate or severe PVR is associated with increased risk of death and heart failure rehospitalizations. Although the incidence of moderate or even severe PVR has declined considerably with the use of second-generation TAVI devices [25], there is evidence that mild PVR, which is still present in a sizable portion of patients, might negatively affect long-term survival as well [1–3] and should be avoided in a low-risk population, in which surgical aortic valve replacement is a viable alternative.

### Clinical implications

We found that annular FD was significantly greater in patients with mild or more PVR than in those with none/trace PVR and that there was a tendency towards higher LVOT FD in patients experiencing our combined endpoint of adverse outcomes, however this difference was not statistically significant. Statistical significance was probably missed because of the relatively low number of included patients.

Although in this analysis, we could not define a threshold above which PVR is to be expected, and FD values of patients with and without mild or more FD are relatively close together, determination of annular FD on individual patient level is useful to gauge PVR risk. In patients with higher FDs, the operator should then specifically aim for optimization of procedural parameters known to be associated with PVR to reduce its probability.

Comparison of predictive performance between FD and known predictors of PVR based on our data is difficult due to relatively low numbers of patients and events and the absence of large trials evaluating PVR predictors in second generation valves. However, the AUC of 0.66 for PVR prediction is roughly of the same magnitude as the AUC of previously published models, which were mostly between 0.635 and 0.72 [7, 21, 26].

Notably, only diastolic but not systolic FD were significantly different between groups. Annulus and LVOT are larger and less elliptical in systole to allow a larger orifice area for blood flow [27]. Probably, the anatomical differences precipitating diastolic paravalvular flow are more subtle in systole, especially in the context of the low absolute differences in FD between patients with and without PVL. However, conventional anatomic predictors of PVR have mostly been found in MDCT during systole [6]. This might be because systolic MDCT is more commonly used for TAVI sizing.

We cannot exclude a small difference in systolic FD becoming more apparent if larger numbers of patients are analyzed. Nonetheless, for PVR prediction, evaluation of diastolic FD seems more useful for future analyses.

In the current analysis, we found a significant difference in FD especially in patients treated with BEV. Although adaptation to a more irregular surface might be worse in BEV than in SEV, the sealing skirt present in second-generation BEV should ameliorate this difference. However, as patient numbers are low, especially when our small population is further split by differentiation of prosthesis type, it is likely that this difference in predictive performance is a chance finding. Further analyses in larger patient groups are necessary to determine whether there is a difference in predictive performance of FD for different prosthesis designs.

### Further steps

As automated segmentation of images for FD determination has been shown to be more reliable [28], we chose to use a custom-build computer program for semiautomated detection of the border line and automated box counting. However, to clinically use the algorithm beyond this proof-of-concept study, the segmentation algorithm must be improved to work with lower luminal contrast filling, which should make automated FD analysis feasible in almost all CT images.

Applicability of the concept to newer-generation valves and other valve designs remains to be elicited. However, as a “difficult anatomy” poses some kind challenge to most valve designs, and FD is a quantitative means of expressing difficulty, it is conceivable that the results are similar.

Most importantly, reevaluation and validation of FD in a large cohort is necessary.

### Limitations

This study has several limitations. First, patient numbers in each group are rather low, mainly because of the strict inclusion criteria for MDCT images owed to the limited capabilities of the segmentation algorithm and the lacking standardization of the images for cardiac phase, drastically reducing statistical power. Second, valid regression analysis was not possible due to the low event number, making it necessary to use the statistical surrogate of comparing FD in different groups rather than its inclusion in a multivariate analysis of the causes of adverse events. Third, the study cohort comprised medium-to-high-risk patients, and it is possible that effects differ in a low-risk cohort.

## Conclusion

In conclusion, this proof-of-concept study shows evidence that FD might be a useful anatomical predictor of adverse events after TAVI. Further investigation of this approach using larger numbers of patients and optimized analysis algorithms to gain a better understanding of the determinants of FD and its predictive performance concerning different clinical endpoints is warranted.

## Supplementary Information

Below is the link to the electronic supplementary material.

Supplementary file1 (PDF 29 kb)
