# Supplementary material for: Fractal dimension of the aortic annulus: a novel predictor of paravalvular leak after transcatheter aortic valve implantation
Source: Int J Cardiovasc Imaging. 2022 Jun 24;38(11):2469–78. doi: 10.1007/s10554-022-02657-1 (PMC9700572; doi:10.1007/s10554-022-02657-1)
Supplement: Supplementary file 1 — Supplementary file1 (PDF 29 kb) [file 10554_2022_2657_MOESM1_ESM.pdf]

Supplementary Data

Stachel et al.: Fractal dimension of the aortic annulus: a novel predictor of paravalvular leak after transcatheter aortic valve implantation

Supplementary Table 1

		Composite endpoint not reached at 30 days	Composite endpoint reached at 30 days	p=
All prosthesis types				
	FD_annulus_diastolic	1.0381 (1.0208-1.0637)	1.0510 (1.0253-1.0606)	0.278
	FD_LVOT_diastolic	1.0394 (1.0229-1.0539)	1.0482 (1.0260-1.0672)	0.432
	FD_annulus_systolic	1.0398 (1.0220-1.0616)	1.0301 (1.0141-1.0560)	0.282
	FD_LVOT_systolic	1.0368 (1.0202-1.0569)	1.0510 (1.0286-1.0611)	0.100
BEV				
	FD_annulus_diastolic	1.0382 (1.0230-1.0634)	1.0342 (1.0261-1.0522)	0.836
	FD_LVOT_diastolic	1.0463 (1.0233-1.0537)	1.0416 (1.0082-1.0450)	0.296

	FD_annulus_systolic	1.0399 (1.0198-1.0598)	1.0268 (1.0188-1.0560)	0.487
	FD_LVOT_systolic	1.0394 (1.0184-1.0609)	1.0554 (1.0508-1.0584)	0.207
SEV				
	FD_annulus_diastolic	1.0331 (1.0208-1.0637)	1.0554 (1.0248-1.0731)	0.180
	FD_LVOT_diastolic	1.0374 (1.0225-1.0561)	1.0569 (1.0360-1.0691)	0.153
	FD_annulus_systolic	1.0395 (1.0249-1.0616)	1.0357 (1.0110-1.0815)	0.449
	FD_LVOT_systolic	1.0349 (1.0218-1.0560)	1.0437 (1.0284-1.0677)	0.269

Supplementary table S1.

Title: FD in a composite endpoint of all-cause death, stroke, moderate or severe PVR and permanent pacemaker implantation

Caption: FD is compared in patients reaching a composite endpoint at 30 days. Data are presented median (IQR).

Supplementary Table 2

	FD_annulus_diastolic	
	Pearson correlation coefficient	p=
LVOT eccentricity	0.225	0.123
LVOT nontubularity	-0.149	0.157
Annulus eccentricity	0.337	0.001
Annulus distensibility	-0.098	0.517
LVOT calcium	0.054	0.607
LVOT calcium density	0.031	0.767
LVOT eccentricity of calcium	0.069	0.604
LVOT NCC calcium	0.120	0.252
LVOT calcium semiquantitative	0.207	0.045
Annulus calcium	0.143	0.169
Annulus calcium density	0.096	0.356
Annulus NCC calcium	0.136	0.192
Aortic valve calcium	0.016	0.882
Aortic valve NCC calcium	-.030	0.775
Aortic valve RCC calcium	-0.020	0.851

Aortic valve LCC calcium	0.071	0.497
Aortic valve calcium semiquantitative	0.009	0.932

Supplementary table S2.

Title: Correlation between FD and conventional anatomic measures

Caption: NCC, non-coronary cusp of the aortic valve, RCC, right-coronary cusp, LCC, left-coronary cusp.
